# Organ preservation strategy: new therapeutic alternative in rectal cancer

**DOI:** 10.1186/s43046-023-00169-0

**Published:** 2023-04-24

**Authors:** F. Safini, B. Amaoui, S. Semghouli, N. Aqodad

**Affiliations:** 1 Radiotherapy Department, Regional Center of Oncology, Agadir, Morocco; 2 Faculty of Medicine and Pharmacy, Radiotherapy Department, University Ibn Zohr, Agadir, Morocco; 3 Higher Institute of Nursing Professions and Health Techniques Agadir, Agadir, Morocco; 4 Faculty of Medicine and Pharmacy, Gastroenterology Department, University Ibn Zohr, Agadir, Morocco

**Keywords:** Organ preservation, Rectal cancer

## Abstract

**Background:**

The therapeutic modalities for nonmetastatic rectal cancer are presently undergoing major changes. The standard treatment is multidisciplinary, combining radiotherapy, chemotherapy, and surgery. The aim of this minireview is to provide an update on the place of organ preservation in the treatment of nonmetastatic rectal cancer in 2022.

**Main text:**

The multimodal strategy based on initial radiochemotherapy followed by radical surgery with excision of the mesorectum has improved oncological results but at the expense of morbidity and sequelae altering life quality. The strategy of rectal preservation has been proposed since the 2000s after the publication of the results of the Brazilian study that proposed a simple surveillance after radiochemotherapy without surgery in good responders. In fact, preoperative radiochemotherapy was able to obtain a complete histological response in 10 to 30% of case. In view of this non-negligible percentage of tumor sterilization, which may well increase with the standardization of total neoadjuvant treatment, a strategy of organ preservation can be proposed in these patients to avoid morbidity and postoperative sequelae.

**Short conclusion:**

This nonoperative approach is currently widely studied in certain patients who have a complete response (clinical, endoscopic, and radiological). However, the selection of these patients is not simple and still complex.

## Background

Over the last few years, rectal cancer has benefited from enormous therapeutic progress in surgery (total mesorectal excision (TME), inter-sphincter surgery); radiotherapy (intensity modulation, contact radiotherapy); and chemotherapy (induction, consolidation, adjuvant chemotherapy). Consequently, the therapeutic recommendations for locally advanced (T3–T4 or N +) nonmetastatic lower and middle rectal cancer continue to evolve.

The distinctive feature of rectal cancer, compared to colon cancer, is that it exposes, in addition to metastatic spread, to very painful local recurrences that are generally unresectable.

In recent years, progress has been made in surgery and in complementary treatments (radiotherapy, chemotherapy). In spite of the 2019 coronavirus (Covid-19) pandemic, the year 2020 has seen promising new data from randomized trials in the field of rectal preservation.

## Materials and methods

We performed this literature review using the PubMed search engine to identify the main articles that reported the place of conservative treatment in the management of rectal cancer.

The MeSH terms used for the search were [preservation OR nonoperative OR nonsurgical] AND [rectal] AND [cancer OR tumor OR neoplasm] AND [treatment OR management].

The search was limited to full-text articles. The selection of articles was based first on the content of the abstracts and then on the content of the full text and clinical relevance.

## Results

### What is the standard treatment for nonmetastatic rectal cancer in 2023?

The treatment of locally advanced rectal cancer tumors (i.e., classified as cT3–T4 and/or N + on MRI) has evolved over the last two decades with the development of a radiosurgical strategy consisting of preoperative chemoradiotherapy (conventional radiotherapy with dose of 50 Gy in 25 sessions over 5 weeks with concomitant administration of an oral chemotherapy of the capecitabine type) or short exclusive radiotherapy of 25 Gy in five fractions followed 6 to 8 weeks later by radical surgery total mesorectal excision [1, 2]. This radiosurgical treatment has reduced the risk of local recurrence to less than 5%, but the risk of metastatic recurrence remains high [3]. In order to reduce the risk of metastatic disease and improve local response, several trials have been conducted using either adjuvant, induction, or consolidation chemotherapy.

The rationale for adjuvant chemotherapy was based on an analogy with the therapeutic indications for colon cancer. Although the Korean phase 2 study (ADORE) demonstrated a benefit of FOLFOX-based adjuvant chemotherapy in ypN + patients, the relevance of adjuvant chemotherapy has never been formally demonstrated in patients undergoing surgery for rectal cancer after radiochemotherapy [4, 5].

Indeed, since the presentation of the results of two randomized trials founding and defending total neoadjuvant therapy (TNT) at ASCO 2020, dogmas have changed, and concomitant radiochemotherapy or short radiotherapy alone should no longer be the standard.

The RAPIDO (Rectal Cancer And Preoperative Induction Therapy Followed by Dedicated Operation) trial is a phase 3 trial that compared total neoadjuvant treatment with short-course radiation therapy (25 Gy, 5 × 5) followed by consolidation chemotherapy such as FOLFOX (oxaliplatin, 5-fluorouracil, folinic acid) or Capox (capecitabine, oxaliplatin) for 4 months and then followed by surgery to standard treatment with preoperative radiotherapy and surgery, optionally followed by adjuvant chemotherapy. The cumulative 3-year probability of disease-related treatment failure was significantly decreased in the experimental group (23.7% vs. 30.4%; *HR* = 0.75 [0.60–0.95]; *p* = 0.019), related to a decreased cumulative 3-year risk of developing distant metastases (20.0% vs. 26.8%, *HR* = 0.69 [0.54–0.90]; *p* = 0.0048) [6].

The randomized phase 3 PRODIGE 23 trial also compared total neoadjuvant therapy (TNT) combining induction chemotherapy based on FOLFIRINOX for 3 months followed by a combination of radiochemotherapy followed by surgery and adjuvant chemotherapy to standard treatment (CAP50 chemoradiotherapy). Oncologic outcomes at 3 years favored the TNT group with an improved probability of disease-free survival (76% vs. 69%; *HR* [hazard ratio] = 0.69 [0.49–0.97]; *p* = 0.034) and an improved probability of metastasis-free survival (78.8% vs. 71.7%; *HR* = 0.64; *p* = 0.017) without toxicity increase [7].

The two regimens evaluated in these trials are very different but have demonstrated that TNT significantly reduces metastatic risk and improves histologic response. Currently in 2023, TNT followed by radical surgery are the standard of care for nonmetastatic locally advanced rectal cancer. For stage T1–T2N0M0 rectal cancer, still surgery with or without chemoradiation in specific cases is the standard of care.

#### The organ preservation strategy in the management of nonmetastatic rectal cancer

##### Why?

In one hand, radiosurgical treatment reduces the risk of local recurrence but at the cost of significant morbidity and functional anorectal, urinary, and sexual sequelae. These sequelae have a negative impact on the quality of life of the patients, especially for tumors of the lower rectum where permanent stomas are often used [8].

On the other hand, preoperative radiochemotherapy has been shown to achieve a complete histological response (pCR) in 10 to 30% of cases depending on the study. This pCR is a good prognostic marker for local and metastatic risk [9].

In view of this non-negligible percentage of tumor sterilization, which is likely to increase with the standardization of TNT, the place of radical surgery in patients with a good response is questioned, hence the consideration of proposing an organ preservation strategy in these patients to avoid morbidity and postoperative sequelae.

##### How?

Several strategies for rectal preservation have been used and proposed.

##### The pure “watch-and-wait” strategy

It is described for the first time by the Brazilian team of A. Habr-Gama in 2004 in a retrospective study of two-hundred and sixty-five patients with resectable adenocarcinoma of the lower and middle rectum (0–7 cm from the anal margin). The patients were treated with radiochemotherapy (external radiotherapy at a dose of 50.4 Gy with concomitant chemotherapy based on 5 FU/Leucorin). Eight weeks after the end of chemoradiotherapy, the evaluation was clinical, radiological, and endoscopic. A biopsy was performed in all patients to evaluate the histological response. It was a simple biopsic sampling of the tumor bed during rectoscopy. Patients with an incomplete response underwent radical surgery, while those with a complete clinical response underwent a nonoperative treatment called the “wait-and-watch” strategy. Patients with a complete response were strictly followed up (clinical examination, digital rectal examination, rectoscopy, and CEA assay every month for 12 months). During the second and third years after treatment, patients were invited to follow-up visits every 2 and 6 months, respectively. Abdominal, pelvic CT scan was repeated every 6 months for the first year. Overall survival and disease-free survival at 5 years were 88% and 83% in the surgical group and 100% and 92% in the watch-and-wait group, respectively [10].

In 2013, the same team published results on the impact of salvage surgery in patients who progressed locally after a watch-and-wait strategy. Recurrences occurred within the first 12 months of follow-up in more than half of cases. A total of 90% of recurrences were treated by surgery allowing good local control of the disease in 94% of patients, with 78% of organ preservation [11].

A meta-analysis published in 2017 analyzed individual data from twenty-three studies that included 867 patients who received a watch-and-wait approach, with a median follow-up ranging from 12 to 68 months. Of note, the majority of studies were retrospective. The choice of the watch-and-wait strategy was often made either after refusal of surgery by the patient or in case of comorbidities contraindicating surgery or following the patient’s choice after discussion with the surgeon. Radiochemotherapy regimens were similar among the included studies. In radiotherapy, the classical protocol was the most used (45 to 50.4 Gy in 25 to 28 fractions); the short protocol was rarely prescribed. Concomitant chemotherapy was based on fluoropyrimidines exclusively (oral capecitabine or bolus fluorouracil).

In this meta-analysis, 15.7% of patients who received a watch-and-wait strategy developed local recurrence but were overtaken by surgery in 95.4% of cases. In eight studies, there was no significant difference between patients with a complete clinical response who received a preservation strategy versus patients with a complete response who underwent radical surgery in terms of local recurrence, cancer-specific mortality, disease-free survival, and overall survival [12].

The other argument in favor of preservation is that local recurrence after WW surgery is possible, whereas local recurrence after radical surgery represents a definitive failure with little potential for recovery. Indeed, only 20 to 30% of patients with locally recurrent rectal cancer after radical surgery will be able to undergo potentially curative R0 resections, whereas after simple surveillance, salvage surgery was possible in more than 90% of cases. This may suggest that salvage surgery would still be effective, and that there was no oncologic risk in offering careful surveillance [13].

However, the main drawbacks of this strategy are the lack of randomized controlled trials, as the majority of published studies are performed in specialized centers that have established individual protocols. There is also a lack of consensus on the definition of a complete clinical response and the need for long-term follow-up. Although several teams have adopted this preservation strategy, the results are not homogeneous, especially in terms of local recurrence at 12 months varying from 5 to 60% [14].

In view of these data, the watch-and-wait strategy remains attractive, but better selection of patients who will benefit from it is necessary.

##### Trans-anal local excision of the scar or tumor residue

This second approach consists in proposing local excision of the scar or tumor residue in patients with a good clinical response after chemoradiotherapy. The advantage of this technique is to have a precise evaluation of the response by an anatomopathological study of the excision specimen and also to include patients with a sub-complete clinical response. However, this preservation strategy has only been proposed for small locally advanced tumors [15].

The American ACOSOG Z6041 trial was the first to suggest that neoadjuvant radiochemotherapy followed by local excision could be considered as a therapeutic alternative to radical surgery in well-selected patients. This phase 2 non-randomized multicenter study focused on small localized tumors less than 4 cm in size classified as T2N0, occupying less than 40% of the circumference of the rectal wall and less than 8 cm from the anal margin [16].

Other prospective trials have evaluated this strategy, but only the French randomized phase 3 GRECCAR 2 trial compared local excision to surgery with total excision of the mesorectum in patients in good response status after chemoradiotherapy defined by a tumor residue ≤ 2 cm for tumors initially classified as cT2 or T3 N0–1, less than 4 cm in size. It is a superiority trial on a composite endpoint (death, local or distant recurrence, morbidity, and side effects at 2 years after surgery). In the excision group, patients with a good response (ypT0–1) received simple surveillance, while those with a poor pathological response (ypT2–3 or R1) underwent total mesorectal excision surgery. This study included 145 patients (74 in the rectal preservation group and 71 in the standard group). In the preservation group, 39 patients had a residual ypT0–T1 lesion and received surveillance. Among the other patients, 27 had a complementary proctectomy, and 8 were not operated for different reasons. This study was negative on the primary endpoint because there was no difference between the two groups on the number of composite endpoint events. However, oncologically, this study demonstrated that the preservation strategy is safe as there was no difference between the two groups on local recurrence rate (7% vs. 7%, *p* = 0.95), recurrence-free survival (70% vs. 73%, *p* = 0.73), and overall survival (84% vs. 82%, *p* = 0.75) [17].

The main disadvantage of this strategy of local excision is related to the morbidity generated by proctectomy on an excisional scar on an irradiated and fragile mucosa, which compromises the potential advantages of preservation. It is also often difficult to differentiate between a postoperative scar and a recurrence, so to avoid unnecessary salvage surgery after excision; better patient selection is needed [13].

### How can we improve the results of rectal preservation?

The data from the International Watch and Wait Database (IWWD), an international multicenter registry, highlights the potential risk of distant metastatic after local regrowth in WW patients. A total of 1009 patients were submitted to the registry between 2015 and 2017 and were treated with a WW approach. At a median follow-up of 3.3 years, the 2-year cumulative incidence of local regrowth was 25.2%. Distant metastases developed in 71 patients (8%) during follow-up. Among the patients with local regrowth, the proportion of distant metastases was higher (38 of 213, 18%) [18].

To improve the results of the preservation strategy, some propose to optimize and intensify the neoadjuvant treatment. This intensification may involve either chemotherapy or radiotherapy.

#### Chemotherapy intensification

Several trials have been conducted to evaluate the impact of chemotherapy intensification. The GRECCAR 12 trial (NCT02514278) is being evaluated to improve the outcomes of rectal preservation strategies. Based on the results of the GRCCAR 2 trial, the GRECCAR 12 investigators have added induction chemotherapy to locally remove tumor residue and increase the rectal preservation rate. The objective of this study was to compare 2 therapeutic strategies, assuming superiority of neoadjuvant chemotherapy and radiochemotherapy over radiochemotherapy alone. This trial included patients with a tumor of the middle and lower rectum classified as cT2–T3 N0–N1 < 4 cm who were randomized to receive either induction chemotherapy (4 cycles of FOLFORINOX) followed by radiochemotherapy or standard radiochemotherapy alone. Evaluation is done between 8 and 10 weeks after the end of treatment by clinical examination and pelvic MRI. Local excision is proposed in good responders (tumor residue ≤ 2 cm), while poor responders underwent radical surgery. The primary objective is the rectal preservation rate at 1 year. Inclusions have been completed, and results are expected in 2022 [15].

Another prospective single-center study conducted by the Brazilian team from Sao Paulo had evaluated extended consolidation chemotherapy after conventional radiochemotherapy. They included seventy patients with a tumor of the distal rectum classified as T2–4 N0–2M0 who received radiochemotherapy (radiotherapy at a dose of 54 Gy + concomitant 5-fluorouracil/leucovorin) followed by consolidation chemotherapy up to 6 cycles every 21 days. Evaluation was performed at 10 weeks after the end of radiotherapy. A complete clinical response was achieved in 68% of patients. After a follow-up of 56 months, the local recurrence rate was 10%, and surgery was avoided in 50% of patients [19].

The OPRA trial was the first randomized trial to evaluate the safety and efficacy of the combination of watch-and-wait and total neoadjuvant therapy (TNT). This phase 2 trial included 324 patients with stages 2 and 3 rectal adenocarcinoma (but the majority were cT3 and cN +) who were randomized to receive 4 months of chemotherapy (FOLFOX or CAPOX) either before (induction) or after (consolidation) conventional radiochemotherapy. Evaluation is performed at 8–12 weeks after the end of treatment by clinical examination, rectosigmoidoscopy, and pelvic MRI. Patients with a complete or subcomplete response were candidates for a WW strategy. The 3-year disease-free survival and metastasis-free survival rates were comparable between the 2 groups (77% vs. 78%, *p* = 0.90 and 81% vs. 83%, *p* = 0.86, respectively), and the 3-year organ preservation rate was significantly higher in the consolidation group (58% vs. 43%, *p* = 0.01) [20].

#### Radiotherapy intensification

Dose escalation of radiotherapy is the second possible option to increase the complete clinical response rate, either by external dose supplementation or by endocavitary irradiation. Endocavitary irradiation has the advantage of delivering additional dose directly to the tumor, by intrarectal introduction of a contact-therapy applicator or a high-dose-rate brachytherapy probe [15].

##### Contact therapy

In France, Professor J. Papillon with the Lyon team had used this technique since the 1970s, publishing a series of 186 patients with small tumors classified T1N0 treated by contact X-ray brachytherapy (CXB) alone and in whom a complete response was obtained in more than 90% of patients. Since the mid-1980s, and based on these results, the same team in Lyon and that of Professor J. P. Gérard in Nice have used CXB as an adjunct to external beam radiotherapy (EBRT) to increase the dose in inoperable elderly patients with T2–3 tumors. Since the 1990s, CXB has been progressively abandoned as the Philips RT 50™ contact machine was no longer manufactured and also with the development of interventional techniques in endoscopic treatment which became the standard treatment for small T1N0 rectal lesions. In 2009, a new machine called Papillon 50™, which produces low-energy 50-kV X-rays, was introduced in the UK and France, marking the renaissance of this technique [21].

In 2012, the Lyon R 96–02 randomized trial proved that compared to neoadjuvant external beam radiotherapy alone, a CXB boost combined with EBRT increased rectal preservation rates. Since then, several centers, especially in France, have continued to evaluate this technique [22].

In 2019, J. P. Gérard et al. published a retrospective analysis of a prospective cohort of patients with distal rectal tumors T2–3N0 < 5 cm treated at three French centers with contact therapy in addition to radiochemotherapy. The 3-year local recurrence rate was 10%, and the cancer-specific survival was 88%. Conservation was possible in 96% of patients. The authors conclude that this approach could be proposed even to operable patients as part of the organ preservation strategy [23].

Based on these encouraging oncologic and functional results, a multicenter randomized phase III OPERA trial (NCT02505750), whose preliminary results on the feasibility of rectal surgery after dose escalation have just been reported and published, is testing the hypothesis that endocavitary boost by contact therapy combined with chemoradiotherapy can increase the rectal preservation rate. This trial included 144 operable patients with T2–T3N0 < 5 cm adenocarcinoma. The standard treatment arm received chemoradiotherapy at a dose of 45 Gy in 25 fractions associated with concomitant capecitabine and boost by external radiotherapy delivering 9 Gy in five fractions in 1 week. Experimental arm B received the same chemoradiotherapy (“cap 45”) followed (or preceded if T was less than 3 cm) by a contact radiotherapy boost (90 Gy in three fractions in 4 weeks). Tumor response was assessed at week 14 and 24 after the start of treatment (digital rectal examination, endoscopy, and MRI). In case of partial response, radical surgery was proposed. In case of complete clinical response, surveillance was recommended. The primary objective was the 3-year overall survival rate without radical surgery. The 3-year organ preservation rate was significantly improved with contact X-ray boost, especially for patients with tumors smaller than 3 cm, treated with contact X-ray brachytherapy first. According to this results, rectal preservation is an option to be discussed in a multidisciplinary consultation meeting and in well-informed patients with early cT2–cT3 disease [24].

##### Iridium brachytherapy

Neoadjuvant iridium brachytherapy, either interstitial or endocavitary, appears to be effective and well tolerated, but the levels of evidence are low. Therefore, it cannot be routinely recommended. In a population of inoperable elderly patients treated with exclusive radiotherapy for T1 to T4 tumors, brachytherapy (30 Gy, 1 fraction of 10 Gy per week) after external EBRT (40 Gy in 16 fractions) allowed a complete response rate of 86.2% with a 2-year local control rate of 71.5% (80% for T1–T2, 67.1% for T3–4). Toxicities consisted of grades 1 or 2 acute rectitis in all patients within 6–8 weeks of treatment and grade 3 late rectitis in 12% of patients [25].

A Danish phase 2 study evaluated the impact of dose escalation with external beam radiotherapy followed by HDR brachytherapy boost on the quality of life of patients with rectal preservation. After a median follow-up of 5 years, the local relapse rate was 31% (95% *CI* 15–47%), and SG was 85% (95% *CI* 75–97%). Long-term patient quality-of-life assessment was excellent [26].

## Conclusion

The current goal of cancer treatment is not only to improve survival but also to improve the quality of life of patients. Organ preservation in rectal cancer is an attractive strategy that is still under evaluation and can only be considered in well-selected patients. Indeed, this strategy still has many limitations both in the definition of complete response, as there is no perfect correlation between complete clinical response and complete pathological response, and in the monitoring modalities which are not yet standardized.

However, with the encouraging results of TNT and the possibility of escalating the dose of radiotherapy with less toxicity, the omission of surgery is an avenue of research to be explored in order to improve the quality of life of patients. With the revolution in molecular biology, it may be possible to move towards a tailor-made treatment adapted to each patient, thus ensuring personalized treatment. In the meantime, the development of a consensus based on the results of trials and published studies is necessary to standardize practices.

## Data Availability

Not applicable.

## Abbreviations

TME: Total mesorectal excision
Gy: Gray
TNT: Total neoadjuvant treatment
pCR: Pathological complete response
CXB: Contact X-ray brachytherapy
EBRT: External beam radiotherapy
